# Belief in safety and ethicality associated with willingness to undergo electroconvulsive therapy among employees of universities and the other research institution

**DOI:** 10.1002/pcn5.40

**Published:** 2022-09-18

**Authors:** Jiro Takeuchi, Yu Sakagami

**Affiliations:** ^1^ Clinical Epidemiology Hyogo Medical University Nishinomiya Japan; ^2^ Occupational Welfare Division, Agency for Health, Safety and Environment Kyoto University Kyoto Japan

Electroconvulsive therapy (ECT) is more effective and safer for severe major depressive disorder (MDD) than drug therapy, even though the pathophysiological mechanisms of ECT have not been elucidated.^1^, ^2^, ^3^ During the modern era, ECT has been improving and has become more ethical^4^; however, patients tend to avoid ECT because of their misconceptions.^5^ Some people perceive ECT as a punishment based on their impressions gained from the media.^6^, ^7^ The ambivalent impressions about ECT generally held may deter treatment opportunities.^8^ We identified the beliefs regarding ECT of people who have never experienced depression and the factors associated with these beliefs.

We conducted a cross‐sectional study in multiple centers in 2008. Attendees of an occupational mental health lecture conducted by A University, B Research Institute, and C University in Japan participated in this study. Before the lecture, a survey instrument on MDD (Supporting Information: Supplemental Materials 1) that was pre‐designed using the expert panel method was distributed to participants, and they answered the quizzes on the survey. The total number of correct answers served as the quiz score. A high score was considered scoring in approximately the top 50% of the responders on the 5‐item quiz on ECT. Exclusion criteria were having possibility of personal identification, having a history of MDD or depressive mood, and incomplete questionnaires. We conducted a multivariable logistic regression analysis to assess the association between willingness to undergo ECT and other factors. To estimate the adjusted odds ratio (aOR) and 95% confidence intervals (CIs), willingness to undergo ECT was included as the binary dependent variable. The following items were included as independent binary or dummy variables: age; gender; occupation; a high quiz score on ECT knowledge; willingness to consult a psychiatrist or the other doctors and a clinical psycologist in the first help‐seeking for MDD; and belief that ECT is efficacious, safe, and ethical. Analyses were performed using Stata 17.0 software (Stata Corporation). All tests of significance were two‐tailed, and *p*‐values < 0.05 were considered statistically significant.

Of the 320 (100%) participants listening to the lecture who agreed to participate in this study, 94 had incomplete questionnaires, seven did not respond to the quiz, one included information related to personal identification, and 60 had a history of MDD or depressive mood. The final analysis included 181 people (56.6%). Supporting Information: Supplementary Material 2 shows the demographic characteristics of the participants. Supporting Information: Supplemental Materials 3‐1 and 3‐2 show the distribution of the quiz scores. The cut‐off score of five items was set to 3.

We identified the factors associated with willingness to undergo ECT (Table 1). In total, 100 (55.2%) people agreed to ECT. There was a positive association between willingness undergo ECT and consulting a psychiatrist or others in the first help‐seeking if one had MDD, as a response to a fictitious question (aOR, 2.50 [95% CI, 1.11–5.66], *p* = 0.03), the belief that ECT is safe (aOR, 2.85 [95% CI, 1.05–7.76], *p* = 0.04), and the belief that ECT is ethical (aOR, 13.0 [95% CI, 4.67–36.1], *p* < 0.001). There was no association between willingness to undergo ECT and the belief that ECT is efficacious (aOR, 0.77 [95% CI, 0.29–2.03], *p* = 0.60).

**Table 1:** Factors associated with willingness to undergo ECT

	WiIlngness to undergo ECT (*n* = 100)	Willingness not to undergo ECT (*n* = 81)	Crude odds ratio (95% CI)	*p*‐value	Adjusted odds ratio (95% CI)	*p*‐value
Age (years)						
20–29	34 (34.0)	37 (45.7)	Reference		Reference	
30–39	44 (44.0)	32 (39.5)	1.50 (0.78–2.87)	0.23	1.52 (0.57–4.08)	0.40
40–49	20 (20.0)	10 (12.3)	2.18 (0.89–5.30)	0.09	3.22 (0.94–11.3)	0.06
60–69 and older	2 (1.4)	2 (2.5)	1.09 (0.15–8.16)	0.93	5.59 (0.24–128)	0.28
Male	73 (73.0)	46 (56.8)	2.06 (1.10–3.84)	0.02	2.55 (1.02–6.38)	0.045
Occupation						
Researcher	7 (7.0)	7 (8.6)	Reference		Reference	
Technician for research work	20 (20.0)	22 (27.2)	0.91 (0.27–3.05)	0.88	2.24 (0.35–14.4)	0.40
Clerical worker	65 (65.0)	47 (58.0)	1.38 (0.45–4.21)	0.57	2.82 (0.50–15.8)	0.24
Student	7 (7.0)	3 (3.7)	2.32 (0.42–12.9)	0.33	4.19 (0.35–50.4)	0.26
Others	1 (1.0)	2 (2.5)	0.50 (0.04–6.86)	0.60	0.77 (0.03–23.0)	0.88
High quiz score on ECT's knowledge (approximately the top 50% of the population and above)	66 (66.0)	37 (45.7)	2.31 (1.26–4.21)	0.006	1.56 (0.70–3.48)	0.28
You would like to consult a psychiatrist or others in the first help‐seeking if you had major depressive disorder	52 (52.0)	26 (32.1)	2.29 (1.25–4.22)	0.008	2.50 (1.11–5.66)	0.03
The belief that ECT is efficacious	64 (64.0)	25 (30.9)	3.98 (2.13–7.43)	< 0.001	0.77 (0.29–2.03)	0.60
The belief that ECT is safe	53 (53.0)	9 (11.1)	9.02 (4.07–20.0)	< 0.001	2.85 (1.05–7.76)	0.04
The belief that ECT is ethical	76 (76.0)	15 (18.5)	13.9 (6.75–28.8)	< 0.001	13.0 (4.67 − 36.1)	< 0.001

*Note*: Values are expressed in terms of frequency (percentage). All factors were adjusted after conducting multivariate logistic regression analysis, such that each factor indicates an independent value for the adjusted odds ratio.

Abbreviation: CI, confidence interval; ECT, electroconvulsive therapy.

Willingness to undergo ECT was positively associated with the belief that ECT is safe and ethical. From the point of safety, no difference between drug therapy and ECT existed in serious medical events.^9^ Regarding ethicality, many people had a negative impression of the ethicality of ECT considering its history. For example, staff had used it occasionally to punish patients in psychiatric wards.^10^ At present, all patients who need ECT should receive enough information by psychiatrists, rather than being influenced by the media.

The variables “High quiz score on ECT's knowledge” and “The belief that ECT is efficacious” were not associated with willingness to undergo ECT in the results after adjustment. Specifically, both variables were significant, which led to the addition of the variable “The belief that ECT is ethical.” “The belief that ECT is ethical” confounds the relationship between “Willingness to undergo ECT” and “The belief that ECT is efficacious.” However, thoughts about ECT seem to be stigmatized regarding its ethicality. In future, we should clarify stigma toward the treatment of mental illness.

This study has some limitations. First, agreement to ECT might have improved since this research period based on a Recommendation in 2013 (https://journal.jspn.or.jp/jspn/openpdf/1150060586.pdf). Second, we did not validate the questionnaires and the quiz. Improving the safety and ethicality of ECT and mitigating the barriers to psychiatric treatment might lead to better acceptance of ECT.

## AUTHOR CONTRIBUTIONS

Jiro Takeuchi contributed to design; contributed to acquisition and interpretation; drafted the manuscript and critically revised the manuscript; performed statistical analysis; gave final approval; and agrees to be accountable for all aspects of the work ensuring integrity and accuracy. Yu Sakagami contributed to conception and design; contributed to acquisition and interpretation; critically revised the manuscript; gave final approval; and agrees to be accountable for all aspects of the work ensuring integrity and accuracy.

## CONFLICT OF INTEREST

The authors declare no conflict of interest.

## ETHICS APPROVAL STATEMENT

This study was approved by the Ethics Committee of the Kyoto University Graduate School of Medicine (E474).

## PATIENT CONSENT STATEMENT

Informed consent was obtained from all the participants.

## Supporting information

Supporting information

## Data Availability

The data that support the findings of this study are openly available.
