# Supplementary material for: Belief in safety and ethicality associated with willingness to undergo electroconvulsive therapy among employees of universities and the other research institution
Source: PCN Rep. 2022 Sep 18;1(3):e40. doi: 10.1002/pcn5.40 (PMC11114321; doi:10.1002/pcn5.40)

**Supplemental materials 1.** Questionnaire and Quiz on Knowledge of Major Depressive Disorder before the Lecture

1) Please write your affiliation.

2) Regarding your age, please answer the following that apply to you.

1. Teenager
2. 20s
3. 30s
4. 40s
5. 50s
6. 60s and older

3) Regarding your gender, please choose one from the following.

1. Male
2. Female

4) Please choose one of the following for your occupation title.

1. Director or Executive
2. Researcher
3. Technician for research
4. Clerical worker
5. Student
6. Others

(4) Have you ever experienced major depressive disorder?

1. Yes
2. No
3. Don't know

5) If you would have major depressive disorder, which kinds of department would you like to consult?

1. Family medicine
2. General medicine
3. Psychiatrics
4. Occupational or school physician
5. Psychologic counseling
6. Others

6) Please choose one regarding efficacy of drug therapy.

1. I don't think that drug therapy is efficacious.
2. I don't think very much that drug therapy is efficacious.
3. I think to some extent that drug therapy is efficacious.
4. I think that drug therapy is efficacious.

7) Please answer regarding efficacy of electro-convulsive therapy.

1. I don't think that electro-convulsive therapy is efficacious.
2. I don't think very much that electro-convulsive therapy is efficacious.
3. I think to some extent that electro-convulsive therapy is efficacious.
4. I think that electro-convulsive therapy is efficacious.

8) Please answer regarding efficacy of cognitive-behavior therapy.

1. I don't think that cognitive-behavior therapy is efficacious.
2. I don't think very much that cognitive-behavior therapy is efficacious.
3. I think to some extent that cognitive-behavior therapy is efficacious.
4. I think that cognitive-behavior therapy is efficacious.

9) Please answer regarding safety of drug therapy.

1. I don't think that drug therapy is safe.
2. I don't think very much that drug therapy is safe.
3. I think to some extent that drug therapy is safe.
4. I think that drug therapy is safe.

9) Please answer regarding safety of electro-convulsive therapy.

1. I don't think that electro-convulsive therapy is safe.
2. I don't think very much that electro-convulsive therapy is safe.
3. I think to some extent that electro-convulsive therapy is safe.
4. I think that electro-convulsive therapy is safety.

10) Please answer regarding safety of cognitive-behavior therapy .

1. I don't think that cognitive-behavior therapy is safe.
2. I don't think very much that cognitive-behavior therapy is safe.
3. I think to some extent that cognitive-behavior therapy is safe.
4. I think that cognitive-behavior therapy is safety.

11) Please answer regarding ethicality of drug therapy.

1. I don't think that drug therapy is ethical.
2. I don't think very much that drug therapy is ethical.
3. I think to some extent that drug therapy is ethical.
4. I think that drug therapy is ethical.

12) Please answer regarding ethicality of electro-convulsive therapy.

1. I don't think that electro-convulsive therapy is ethical.
2. I don't think very much that electro-convulsive therapy is ethical.
3. I think to some extent that electro-convulsive therapy is ethical.
4. I think that electro-convulsive therapy is ethical.

13) Please answer regarding ethicality of cognitive-behavior therapy.

1. I don't think that cognitive behavior-therapy is ethical.
2. I don't think very much that cognitive-behavior therapy is ethical.
3. I think to some extent that cognitive-behavior therapy is ethical.
4. I think that cognitive-behavior therapy is ethical.

14) If you would be a patient with major depressive disorder, do you agree with drug therapy?

1. I don't agree with drug therapy.
2. I don't agree very much with drug therapy.
3. I agree to some extent with drug therapy.
4. I agree with drug therapy.

15) If you would be a patient with major depressive disorder, do you agree with electro-convulsive therapy?

1. I don't agree with electro-convulsive therapy.
2. I don't agree very much with electro-convulsive therapy.
3. I agree to some extent with electro-convulsive therapy.
4. I agree with electro-convulsive therapy.

16) If you would be a patient with major depressive disorder, do you agree with cognitive-behavior therapy?

1. I don't agree with cognitive-behavior therapy.
2. I don't agree very much with cognitive-behavior therapy.
3. I agree to some extent with cognitive-behavior therapy.
4. I agree with cognitive-behavior therapy.

Please read quiz following sentence and answer with a "〇" in correct and a "×" in incorrect.

1. Physical symptoms such as pain are rare in depression.
2. Antidepressants (drugs for depression) have few side effects.
3. Cognitive-behavioral therapy for depression can be expected to reduce depressive flare-ups.
4. Electro-convulsive therapy may be an option for refractory depression.
5. Medication is a rare treatment for depression.
6. Depression often causes changes in appetite and sleep.
7. Electro-convulsive therapy for depression is often performed under general anesthesia.
8. Cognitive-behavioral therapy for depression is appropriate for severely ill patients.
9. Medications other than antidepressants may be used in the pharmacotherapy of depression.
10. Suicide with depression is most caused at the peak of symptoms.
11. Antidepressants are often effective as soon as they are taken.
12. Rest is the first step in the treatment of depression.
13. Depression is a laziness disease caused by lack of effort.
14. Electro-convulsive therapy for depression is not paid by insurance.
15. Cognitive-behavioral therapy for depression is intensive in the early stages of treatment.
16. Electro-convulsive therapy for depression is appropriate for severely ill patients.
17. Antidepressants are effective in almost all patients.
18. Cognitive-behavioral therapy for depression is often administered by counselors.
19. Electro-convulsive therapy for depression is often used without informed consent by patient or family.
20. Cognitive-behavioral therapy for depression is often used in combination with other treatments.

**Supplemental materials 2.** Characteristics of participants and final analysis population

|  | | Participants (n = 320) | Analysis target population (n = 181) | Not analysis target population (n = 139) | P-value |
| --- | --- | --- | --- | --- | --- |
| Gender (male) (n = 314) | | 189 (60.2) | 119 (65.8) | 70 (52.6) | 0.02 |
| Age (years) (n = 315) | 10 - 19 | 1 (0.3) | 0 (0) | 1 (0.8) | < 0.001 |
|  | 20 - 29 | 97 (30.8) | 71 (39.2) | 26 (19.4) |  |
|  | 30 - 39 | 113 (35.9) | 76 (42.0) | 37 (27.6) |  |
|  | 40 - 49 | 54 (17.1) | 30 (16.6) | 24 (17.9) |  |
|  | 50 - 59 | 44 (14.0) | 0 | 44 (32.8) |  |
|  | 60 - 69 and older | 6 (1.9) | 4 (2.2) | 2 (1.5) |  |
| Affiliation | A | 250 (78.1) | 158 (87.3) | 92 (66.2) | < 0.001 |
|  | B | 62 (19.4) | 20 (11.0) | 42 (30.2) |  |
|  | C | 8 (2.5) | 3 (1.7) | 5 (3.6) |  |
| Occupation (n = 307) | Researcher | 29 (9.5) | 14 (7.7) | 15 (11.9) | 0.53 |
|  | Technician for research | 73 (23.8) | 42 (23.2) | 31 (24.6) |  |
|  | Clerical worker | 181 (58.9) | 112 (61.9) | 69 (54.8) |  |
|  | Student | 20 (6.5) | 10 (5.5) | 10 (7.9) |  |
|  | Others | 4 (1.3) | 3 (1.7) | 1 (0.8) |  |

Values are expressed in terms of frequency (percentage).

**Supplemental materials 3-1. The Distribution of 20-item Quiz Score about Knowledge of Major Depressive Disorder**

Cronbach’s alpha = 0.75


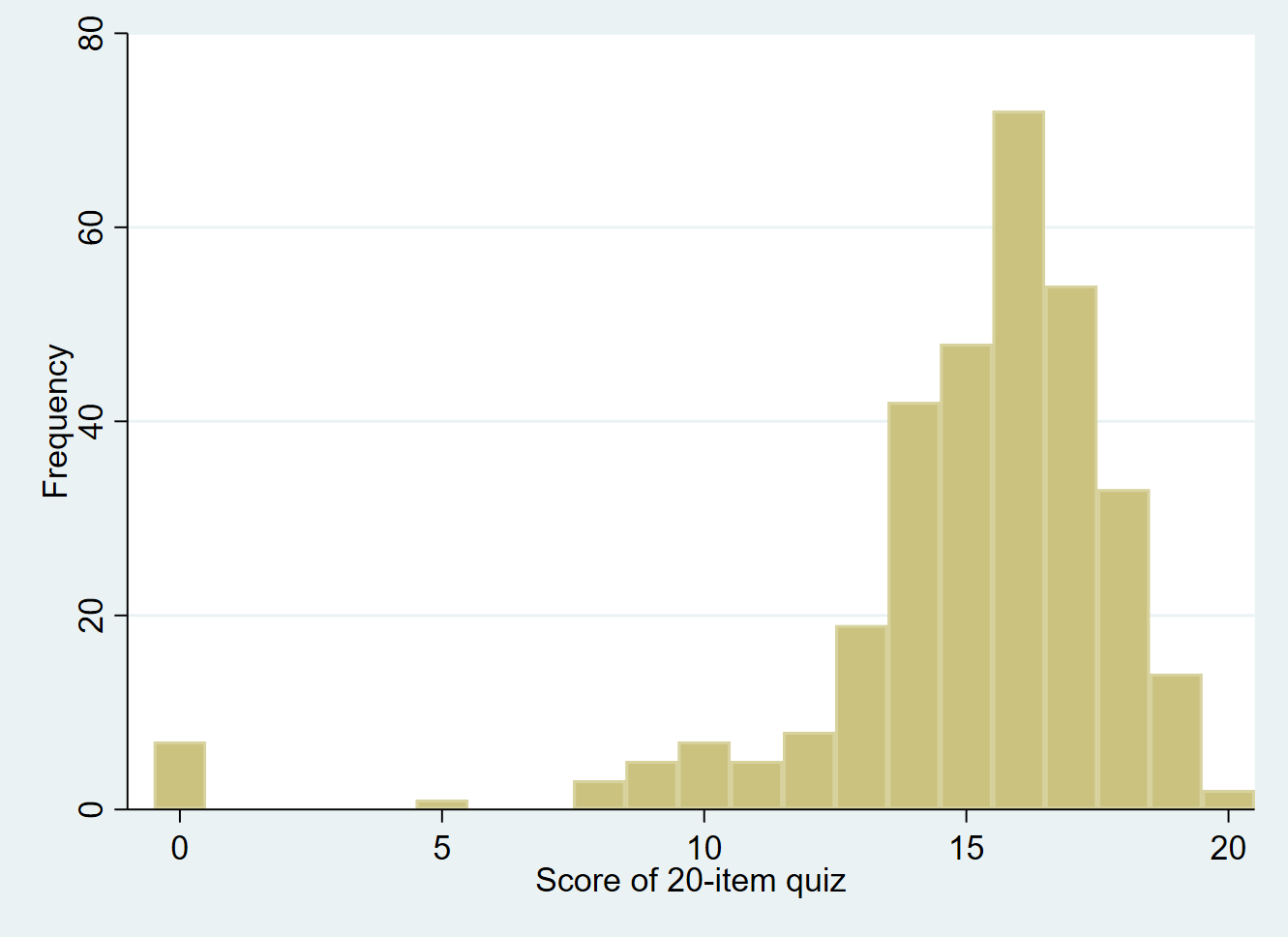


**Supplemental materials 3-2. The Distribution of 5-item Quiz Score extracted from the 20-item Quiz about Knowledge of Electro-convulsive therapy for Major depressive disorder**

Cronbach’s alpha = 0.42


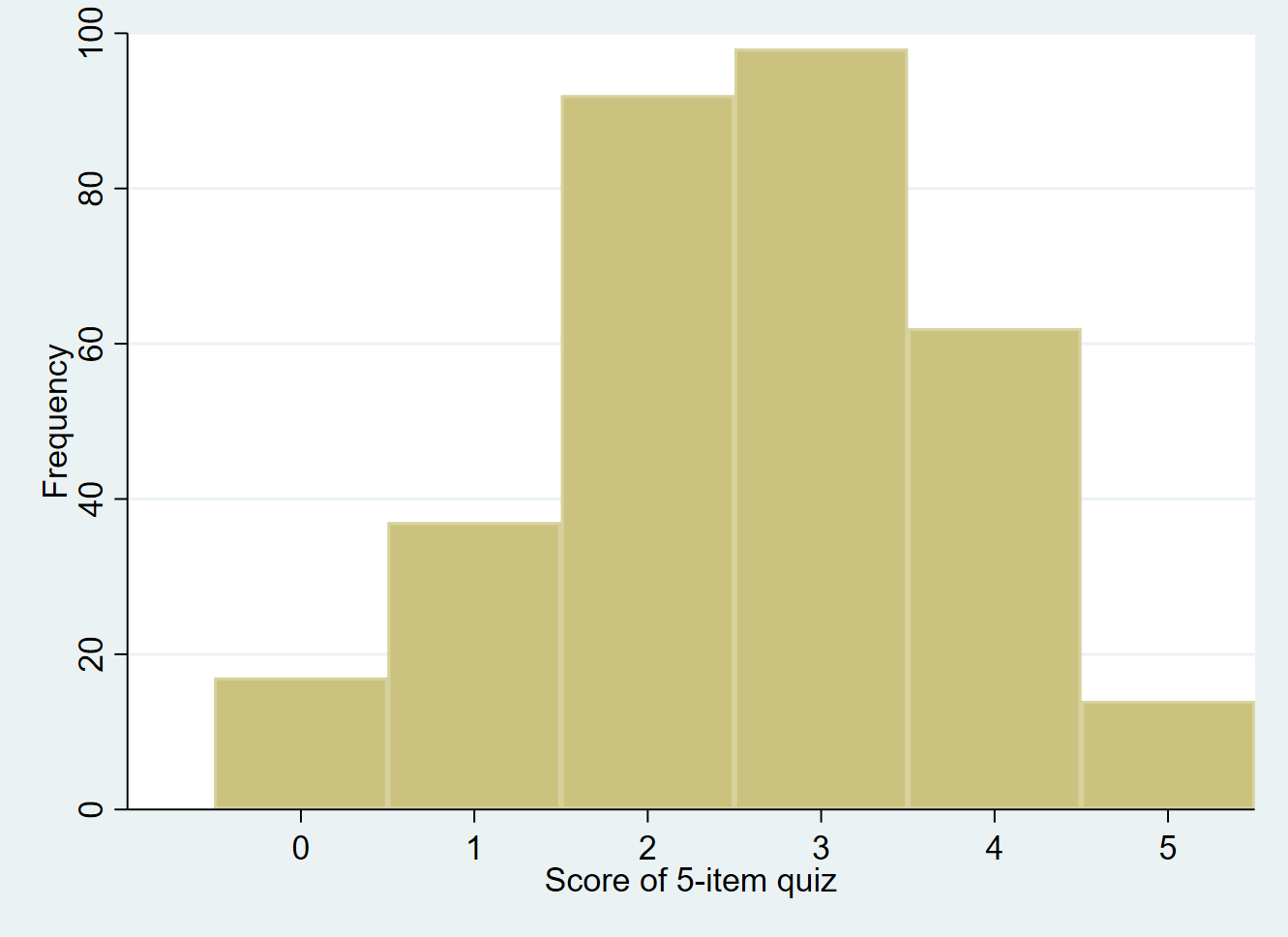

Supplement: Supplementary file 1 — Supporting information [file PCN5-1-e40-s001.docx]
